# Characterization of myocardial edema in rats with acute reperfused myocardial infarction at multiple time points by 7 T MR

**DOI:** 10.1186/1532-429X-18-S1-P65

**Published:** 2016-01-27

**Authors:** Wei Chen, Ziqian Xu, Yushu Chen, Ruzhi Zhang, Jie Zheng, Fabao Gao

**Affiliations:** 1 Depart of Radiology, West China Hospital of Sichuan University, Chengdu, China; 2 Mallinckrodt Institute of Radiology, Washington University School of Medicine, St. Louis, MO USA

## Background

Intramyocardial hemorrhage(IMH) caused by reperfusion after acute myocardial infarction (MI) is considered to be an important independent predictor of adverse left ventricular remodeling and clinic outcomes. We aimed to characterize the evolution of myocardial edema (ME) in rats with acute reperfused MI, and to explore the effect of IMH post reperfusion on ME.

## Methods

MI model was induced on 17 Sprague-Dawley rats (female, 260-280 g) by ligating the left anterior descending or circumflex coronary arteries for 60 minutes, followed by reperfusion. The rats were then scanned in a 7 T MRI system at 24 h, 48 h, 72 h, and 5 d after reperfusion. Area of ME and T2 values of ME were measured on T2 mapping images. Myocardial infarction was validated by the late gadolinium enhancement (LGE) method. IMH was detected by T2w. Repeated measures analysis of variance and independent sample t test were used for statistical analysis.

## Results

**Figure 1:**
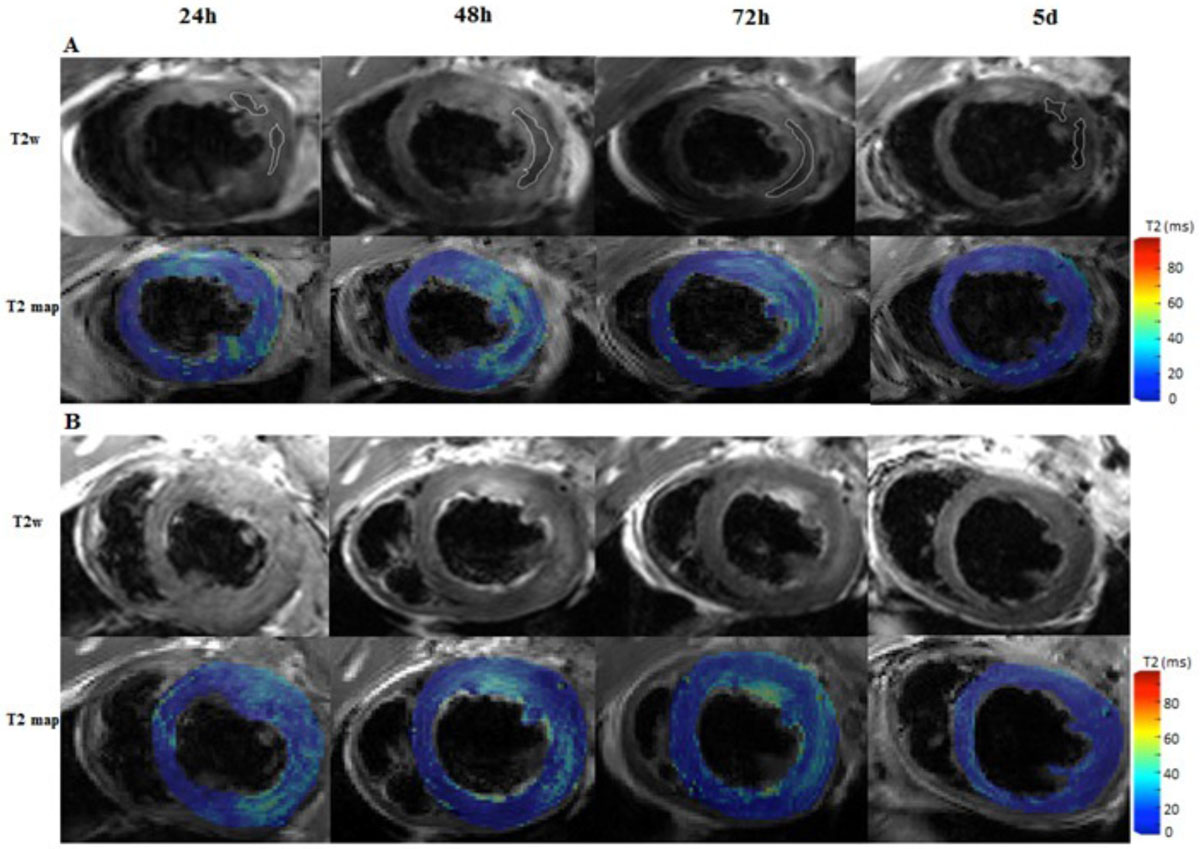
**Cardiovascular magnetic resonance scan of rats with and without intramyocardial hemorrhage**. A, In a rate with intraomyocardial hemorrhage (T2w, white line), T2 map shows increased edema area and T2 value at 48 h, which gradually decreased from 48 h to 5 d. B, In a rate with no hemorrahgem T2 map demonstrates larger edema area and higher T2 value at 24 h, which gradually decreased from 24 h to 5 d. T2w shows no intramyocardial hemorrhage.

**Figure 2:**
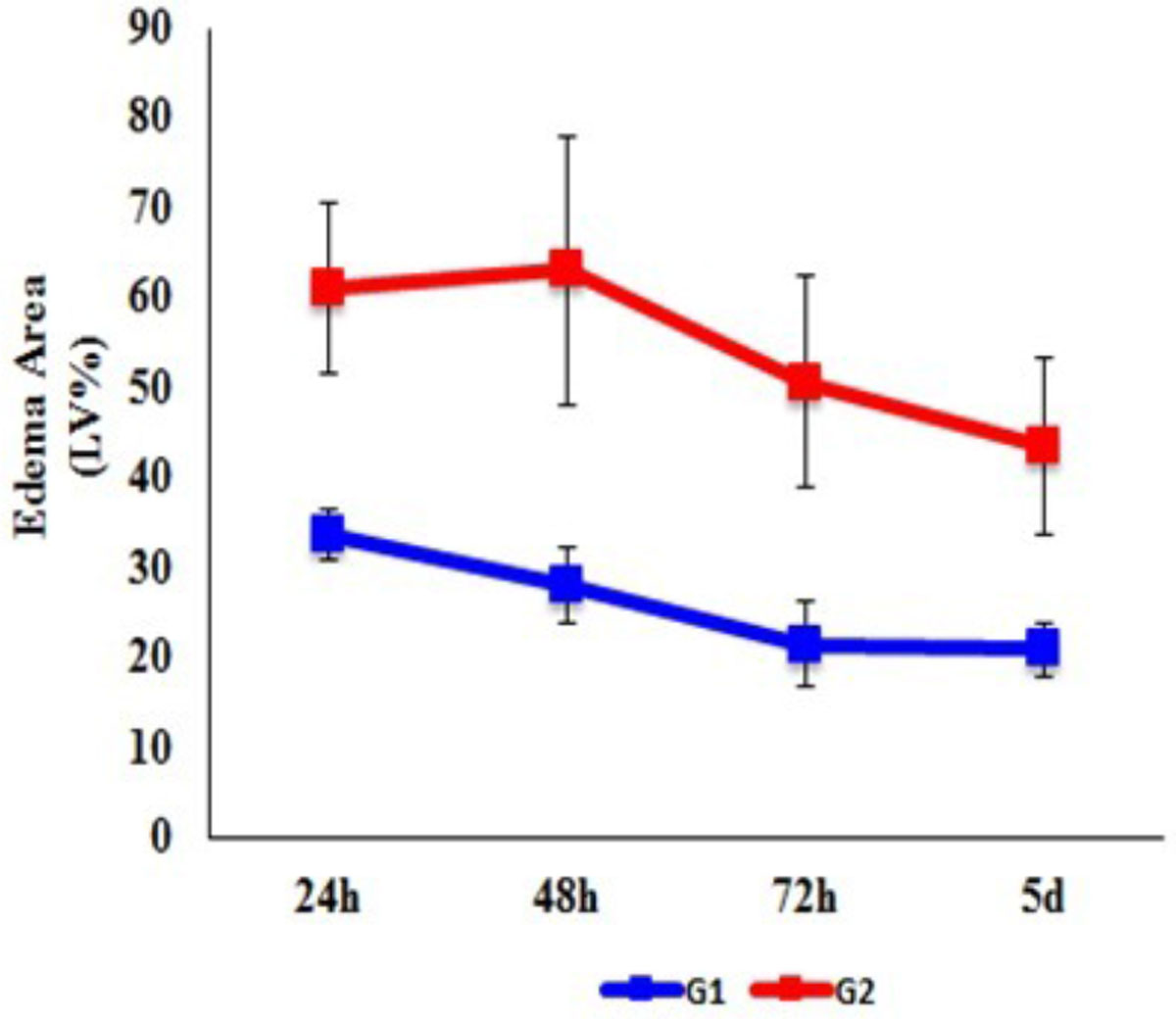
**Evolution of myocardial edema area in G1 and G2 over time**. The edema area in G2 is larger than that in G1 over time. The edema area in G2 peaks at 48 h, which gradually decreases from 48 h to 5 d, whereas the edema area in G1 peaks at 24 h. G1: myocardial infarction without intramyocardial hemorrhage G2: myocardial infarction with intramyocardial hemorrhage.

**Figure 3:**
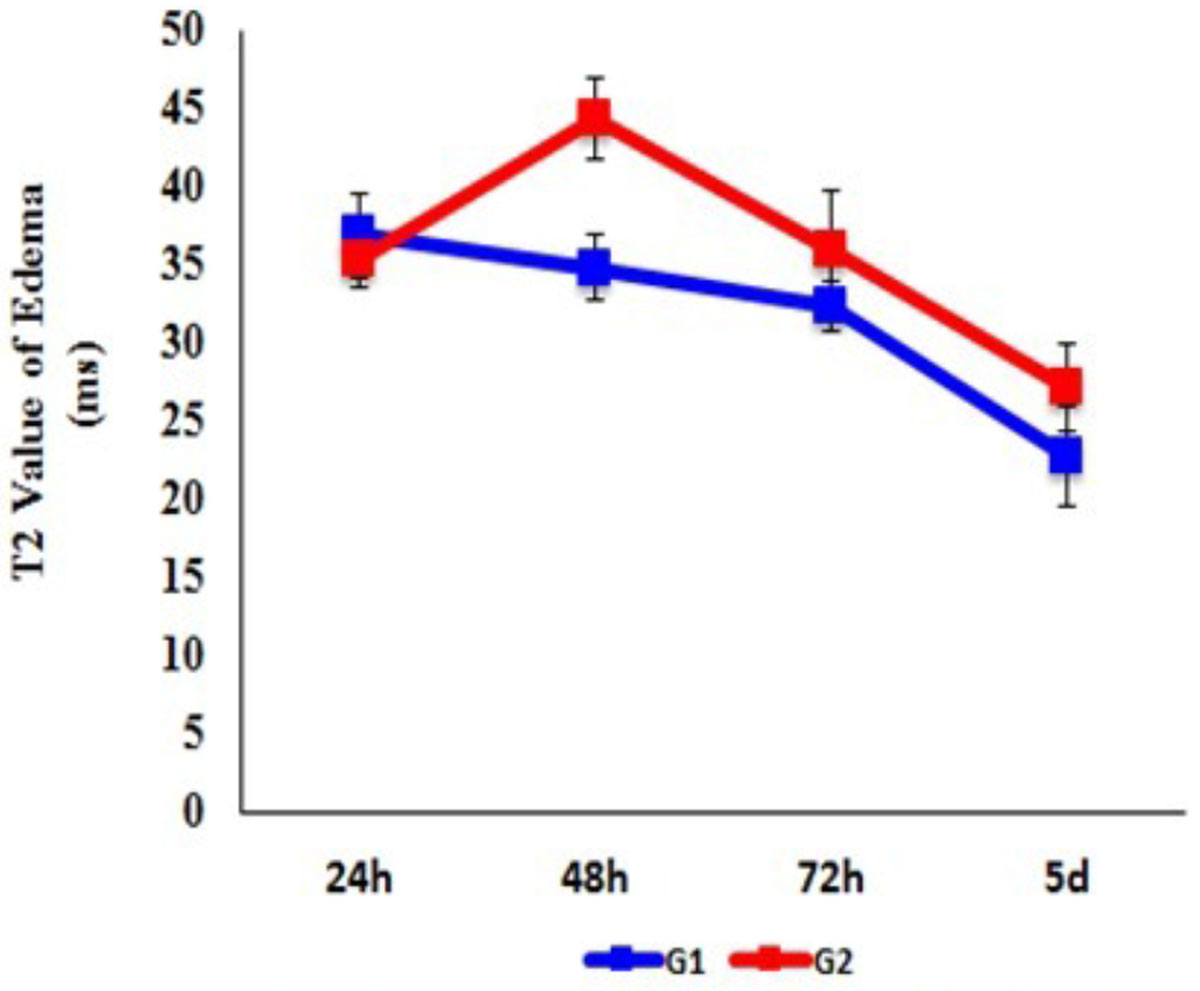
**Evolution of T2 value of myocardial edema in G1 and G2 over time**. There is a tendency for higher T2 values in G2, compared with those in G1. T2 value or myocardial edema peaks at 48j in G2, whereas it peaks at 24 h in G1. G1: myocardial infarction without intramyocardial hemorrhage G2: myocardial infarction with intramyocardial hemorrhage.

9/17 rats died, 8 survived and was divided into two groups, G1(n = 3), MI without IMH, G2(n = 5), MI with IMH, according to the absence or the presence of IMH on T2w at 24 h. Representative T2w and T2 mapping images acquired at 24 h, 48 h, 72 h, and 5 d from one rats with IMH after reperfused MI and one rat without IMH are shown in Figure 1. Area of ME, expressed as the percentage of left ventricular myocardium (LV%), in G1 and G2 at the four time points were: 33.6% ± 2.9%, 28.1% ± 4.1%, 21.5% ± 4.8%, 20.9% ± 3.1%, and 61.0% ± 9.4%, 62.9% ± 15%, 50.6% ± 11.8%, 43.5% ± 9.9%, respectively. Area of ME differed significantly at any other two time points within each group (*p*<0.001 for both group). Area of ME in G2 was significantly larger than that in G1 (*F*=11.476, *p*=0.02) at each time point. The maximum was observed at 48 h, then gradually decreasing from 48 h to 5 d. The area of ME in G1 reached maximum at 24 h, and then decreased in the following time points (Figure 2). T2 values of ME in G1 and G2 at the four time points were: 36.9 ± 2.6 ms, 34.8 ± 2.1 ms, 32.3 ± 1.6 ms, 22.8 ± 3.2 ms, and 35.4 ± 1.9 ms, 44.3 ± 2.5 ms, 36.0 ± 3.8 ms, 27.2 ± 2.8 ms, respectively. T2 values of ME also differed significantly at any other two time points within each group(*p*<0.005 for both group). T2 values of ME in the two group had no significant difference (*F* = 6.27 *p* = 0.054), however, there was a tendency for higher T2 values in G2, compared with those in G1. T2 values of ME in G2 also reached maximum at 48 h, and then gradually decreased. T2 values of ME in G1 were at the highest level at 24 h, and then decreased in the following time points (Figure 3).

## Conclusions

Myocardial edema in rats after acute reperfused myocardial infarction with or without IMH evolves differently over time, hemorrhagic infarction indicates more severe myocardial damages than non-hemorrhagic infarction, with more extent and more severity of myocardial edema.

